# HPV vaccination in Africa in the COVID-19 era: a cross-sectional survey of healthcare providers’ knowledge, training, and recommendation practices

**DOI:** 10.3389/fpubh.2024.1343064

**Published:** 2024-01-17

**Authors:** Joel Fokom Domgue, Issimouha Dille, Sharon Kapambwe, Robert Yu, Freddy Gnangnon, Lameck Chinula, Gad Murenzi, Nomonde Mbatani, Mala Pande, Fatoumata Sidibe, Joseph Kamgno, Bangaly Traore, Hicham El Fazazi, Mamadou Diop, Pierre-Marie Tebeu, Mohenou Isidore Diomande, Fabrice Lecuru, Isaac Adewole, Marie Plante, Partha Basu, Jean-Marie Dangou, Sanjay Shete

**Affiliations:** ^1^Division of Cancer Prevention and Population Sciences, The University of Texas MD Anderson Cancer Center, Houston, TX, United States; ^2^Department of Public Health, Faculty of Medicine and Biomedical Sciences, University of Yaounde, Yaoundé, Cameroon; ^3^Department of Gynecology and Obstetrics, Faculty of Medicine and Biomedical Sciences, University of Yaounde, Yaoundé, Cameroon; ^4^Centre Inter-Etats d'Enseignement Supérieur en Santé Publique d'Afrique Centrale, Brazzaville, Republic of Congo; ^5^Division of Noncommunicable Diseases, World Health Organization Regional Office for Africa, Brazzaville, Republic of Congo; ^6^Division of Surgical Oncology, Faculty of Health Sciences, University of Abomey-Calavi, Cotonou, Benin; ^7^University of North Carolina Project-Malawi, Lilongwe, Malawi; ^8^Division of Global Women’s Health, Department of Obstetrics and Gynecology, University of North Carolina at Chapel Hill, Chapel Hill, NC, United States; ^9^Einstein-Rwanda Research and Capacity Building Program, Rwanda Military Hospital, Kigali, Rwanda; ^10^Department of Pathology, School of Medicine, Muhimbili University of Health and Allied Sciences, Dar es Salaam, Tanzania; ^11^Gynecologic Oncology Unit, Groote Schuur Hospital, Faculty of Health Sciences, University of Cape Town, Cape Town, South Africa; ^12^Medical Oncology Unit, CHU du Point G, Faculty of Medicine and Dentistry, University of Bamako, Bamako, Mali; ^13^Division of Surgical Oncology, Faculty of Health Sciences and Technics, University Gamal Abdel Nasser of Conakry, Conakry, Guinea; ^14^Department of Gynecology and Obstetrics, Faculty of Medicine and Pharmacy, Mohammed V University, Rabat, Morocco; ^15^Institut du Cancer Joliot Curie, CHU Aristide Le Dantec, Dakar, Senegal; ^16^Department of Pathology, University Teaching Hospital of Cocody, Abidjan, Côte d'Ivoire; ^17^Department of Gynecologic and Breast Surgical Oncology, Institut Curie, Paris, France; ^18^Department of Obstetrics and Gynecology, Faculty of Clinical Sciences, College of Medicine, University of Ibadan, Ibadan, Nigeria; ^19^Division of Gynecologic Oncology, Department of Gynecology and Obstetrics, Laval University, Québec City, QC, Canada; ^20^Screening Group, Section of Early Detection and Prevention, International Agency for Research on Cancer, Lyon, France

**Keywords:** HPV, cervical cancer, HPV-related disease, HPV vaccination, vaccine recommendation, vaccine hesitancy, cancer control, healthcare providers

## Abstract

**Introduction:**

Although the burden of cervical cancer in Africa is highest, HPV vaccination coverage remains alarmingly low in this region. Providers’ knowledge and recommendation are key drivers of HPV vaccination uptake. Yet, evidence about providers’ knowledge and recommendation practices about the HPV vaccine against a backdrop of emerging vaccine hesitancy fueled by the COVID-19 pandemic is lacking in Africa.

**Methods:**

A cross-sectional study was conducted in 2021–2022 among healthcare providers involved in cervical cancer prevention activities in Africa. They were invited to report prior training, the availability of the HPV vaccine in their practice, whether they recommended the HPV vaccine, and, if not, the reasons for not recommending it. Their knowledge about the HPV vaccine was assessed through self-reporting (perceived knowledge) and with three pre-tested knowledge questions (measured knowledge).

**Results:**

Of the 153 providers from 23 African countries who responded to the survey (mean age: 38.5 years, SD: 10.1), 75 (54.0%) were female and 97 (63.4%) were based In countries with national HPV immunization programs. Overall, 57 (43.8%) reported having received prior training on HPV vaccine education/counseling, and 40 (37.4%) indicated that the HPV vaccine was available at the facility where they work. Most respondents (109, 83.2%) reported recommending the HPV vaccine in their practice. Vaccine unavailability (57.1%), lack of effective communication tools and informational material (28.6%), and need for adequate training (28.6%) were the most commonly reported reasons for not recommending the HPV vaccine. While 63 providers (52.9%) reported that their knowledge about HPV vaccination was adequate for their practice, only 9.9% responded correctly to the 3 knowledge questions.

**Conclusion:**

To increase HPV vaccination coverage and counter misinformation about this vaccine in Africa, adequate training of providers and culturally appropriate educational materials are needed to improve their knowledge of the HPV vaccine and to facilitate effective communication with their patients and the community.

## Introduction

Human papillomavirus (HPV) – the most common sexually transmitted virus globally - is responsible for cervical cancer, a leading cause of cancer morbidity and mortality among women in Africa, where approximately 415 million women aged 15 years and older are at risk of developing this preventable condition (1). Just like women, men in Africa are exposed to HPV-associated cancers (oropharyngeal, anal, and penile cancers) as well as genital warts (2). The prevalence of HPV infection was reported to be twice as higher in Africa compared to global estimates (22–24% versus 11–12%) (3).

**Figure 1:**
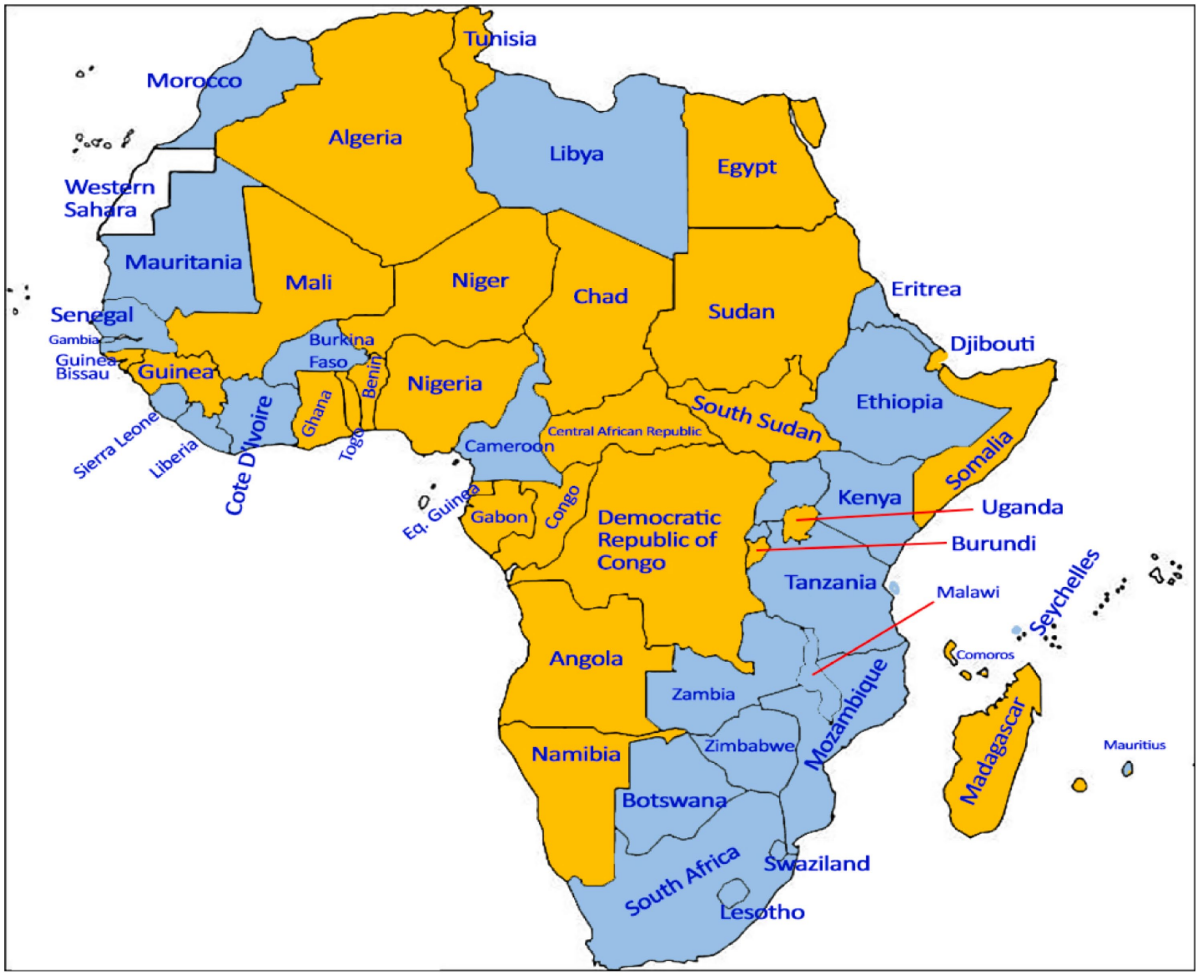
Map of African Countries according to the HPV vaccine national introduction status. 
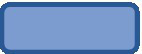
 African countries that had introduced HPV vaccine in their national immunization programs as of 2022. 
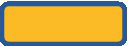
 African countries that had not introduced HPV vaccine in their national immunization program as of 2022. 
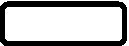
 Data Not available. Adapted from the WHO’s Global Health Observatory: https://www.who.int/data/gho/data/indicators/indicator-details/GHO/existence-of-national-hpv-vaccination-programme.

In 2009, the HPV vaccine was endorsed by the World Health Organization (WHO) for girls aged 9 to 14 to prevent HPV-associated diseases (4). Although in use in the private sector in some urban African settings, HPV vaccines were not introduced in National Immunization Programs (NIPs) until 2011, when Rwanda became the first African country to successfully implement an HPV vaccination program (5). Between 2013 and 2016, GAVI (the Vaccine Alliance) initiated the provision of support to eligible countries (e.g., Zambia in 2013, Malawi in 2013, Uganda in 2015) for 2-year HPV vaccine demonstration projects, and GAVI funding for national rollouts in these countries became available in mid-2016 (6). As of 2022, only 40% (22 out of 54) of African countries had effectively included HPV vaccine in their NIPs (7) (Figure 1).

The HPV vaccination schedule has evolved since the vaccine was approved, from a three-dose regimen recommended by the WHO between 2009 and 2014; to a two-dose regimen (for girls below the age of 15) between 2014 and 2022. Since December 2022, WHO has updated its recommendations in light of new scientific evidence for a cost-effective single-dose HPV vaccine to endorse a one-dose regimen for girls and women below the age of 21 (8). With the reduction in the number of doses, vaccine uptake in limited resource settings is expected to increase even faster through the reduction of system-related barriers such as logistic constraints, workforce shortage, and vaccine prices (9).

While the HPV vaccine has been available for about a decade in some parts of Africa, dose completion in 2022 was as low as 20% (on average) in African countries that introduced the HPV vaccine in their NIP (10). Despite the progress made so far, this rate is far below the WHO target of 90% vaccination coverage to be achieved by 2030 to accelerate the elimination of cervical cancer by the end of the millennium.

The effect of high HPV vaccine coverage on mitigating the burden of HPV-related conditions is now observed in high-income settings where organized HPV immunization programs are widely implemented (11). In a recent report of a cohort of vaccinated girls in Rwanda, a reduction in the prevalence of vaccine-targeted HPV types from 12% (in 2013–2014) to 5% (in 2019–2020) was observed in sexually active women aged 17–29 years (12). Despite this strong evidence, many African countries are still lagging behind in implementing HPV immunization programs in a context where HPV-related morbidity and mortality remain high (13). Further, the growing access to health information through the internet and social media in recent years in Africa, combined with the limited involvement of frontline healthcare providers, has accelerated the spread of vaccine hesitancy in the African population, especially in the COVID-19 era where misbeliefs and misconceptions about vaccines have been exacerbated (14).

While providers’ recommendation has been consistently recognized as a driving factor for HPV vaccine uptake (15), little is known about providers’ practices and recommendation for HPV vaccination in Africa. Further, the low level of knowledge about HPV vaccination which is also recognized as a strong barrier to HPV vaccine acceptance has not been characterized in Africa. In this study, we assessed the training, knowledge, and recommendation of the HPV vaccine among African providers involved in cervical cancer prevention activities. These findings will serve as a basis for developing and evaluating targeted interventions to improve provider communication about the HPV vaccine against a backdrop of increased vaccine hesitancy and increased population access to health information through social media and the internet in Africa.

## Methods

### Study population and study design

The study population consisted of healthcare providers involved in cervical cancer prevention activities from 23 African countries who were enrolled in a distance learning program focusing on cervical cancer and other HPV-related anogenital diseases (16, 17). Between 2021 and 2022, providers were invited to take an online survey (in English or French) to assess their training, knowledge, and practices toward cervical cancer screening, management of pre-invasive lesions, and HPV vaccination (Supplementary Figure S1). The questionnaire was pre-tested and validated before being administered to the target population. This was done in two steps: In a first step, after developing the survey tool, we shared it with 4 experts to get their feedback and remarks regarding content validity. Suggestions from these experts were then incorporated into the survey tool. In a second step, the self-administered survey was pilot tested with a convenience sample of 20 individuals of varying healthcare provider professions based in Africa to ensure clarity of questions and ease of administration. Further comments from this set of HCPs were accounted for in the final revision of the questionnaire. Participation was anonymous and voluntary, and refusal to take the survey had no consequence on participation in the distance learning program. A detailed description of the survey design, content, and administration has been published elsewhere (16).

### Measures

#### Main variable

We assessed whether African-based providers involved in cervical cancer prevention activities were recommending HPV vaccination in their practice using the following questions. “*Do you currently recommend HPV vaccination in your practice?*” If the answer to this question was “yes,” a follow-up question was asked: “*Who do you recommend the HPV vaccine for?*” The possible responses to this follow-up question included: “I recommend HPV vaccine for girls only,” and “I recommend HPV vaccine for both girls and boys.” If the answer to the first question was “no,” the follow-up question was: *“What is/are the reason(s) for not recommending HPV vaccination in your practice?.”* Possible responses to the latter included: “HPV vaccination is not available in my practice,” “I delegate these activities to other staff in my practice,” “Additional training would be needed to perform some of these activities,” “Not enough time,” “Lack of effective tools and information to give to patients,” “Patient/family cannot afford the cost of the HPV vaccine,” “I chose not to perform these activities,” “Not needed because cervical cancer screening is effective,” and “Some other reasons.”

#### Additional variables

Prior training on HPV vaccine counseling was assessed with the following question: “*Have you previously had training in how to educate your patients, their family members, and the population about HPV vaccination?*” (Yes/No). Availability of HPV vaccine was assessed with the following question: “*Is HPV vaccine currently available at the facility where you work?*” (Yes/No).

To better describe the study population, we collected the following socio-demographic variables: age (years), gender (Male/Female), location according to the United Nation’s classification of African regions (Eastern Africa, Middle Africa, Western Africa, Southern Africa, and Northern Africa), and setting (urban/rural). Age was described as a continuous variable. Providers were also classified according to their educational background into doctors (including family medicine physicians, internists, obstetricians-gynecologists, oncologists, pediatricians, surgeons, pathologists, etc.), nurses (including midwives), and non-clinician providers (including health administrators, project managers, and community health workers involved in cervical cancer prevention activities).

### Knowledge questions

#### Measured knowledge

The level of knowledge about HPV vaccination was assessed with three statements to which participants were asked to provide either of the three possible responses: “true,” “false,” or “I do not know.” The first statement was about vaccine efficacy: (i) “*A 13-year-old girl receives two doses of the quadrivalent HPV vaccine. By doing so she expects a 90–100% protection rate against future development of cervical cancer*.” The second statement was about the vaccine administration schedule: (ii) “*A 16-year-old girl has received only one dose of HPV vaccine when she was 13 years old. To complete her vaccination schedule, she should restart HPV vaccination from the first dose*.” The third statement was about vaccine safety: (iii) “*HPV vaccination reduces fertility of women*.”

#### Perceived knowledge

In addition to the measured knowledge, self-reported knowledge about the HPV vaccine was assessed with the following statement: “*My knowledge about HPV vaccination is adequate for my current practice*.” Possible responses to this statement included: “Agree,” “Disagree,” “Neither Agree nor Disagree,” and “I do not know.”

### Statistical analysis

Analyses were performed using statistical analysis software *The SAS* (v9.4). We classified healthcare providers into 2 groups according to the introduction status of the HPV vaccine into the NIP: providers working in countries with national HPV immunization programs and those working in countries without national HPV immunization programs (Figure 1). Descriptive statistics were tabulated in each group. We also examined the gender difference (female providers versus male providers) in HPV vaccine knowledge and recommendation practices. Comparisons between groups were carried out using Fisher’s exact test for categorical variables and the likelihood ratio chi-square test for continuous variables. Missing data were excluded by listwise deletion. Two-sided *p* < 0.05 was considered statistically significant.

This study was conducted according to the strengthening the Reporting of observational studies in epidemiology (STROBE) guidelines (Supplementary Table S1).

#### Ethics approval

This research conformed to the principles embodied in the Declaration of Helsinki. All participants provided written informed consent. The study protocol was approved by the University of Texas MD Anderson Cancer Center’s IRB.

#### Patient and public involvement

Patients or public were not involved in the design, or conduct, or reporting, or dissemination plans of our research.

## Results

### Characteristics of the study population

Of the 153 (mean [SD] age, 38.5 [10.1] years) respondents who completed the survey, most were female (75 [54.0%]) and working in urban areas [113 (89.7%)]. Participants were from 23 African countries, including 16 providers (10.5%) from Eastern Africa, 64 (41.8%) from Middle Africa, 10 (6.5%) from Northern Africa, 6 (3.9%) from Southern Africa, and 57 (37.3%) from Western Africa (Table 1).

**Table 1:** Characteristics of the study population.

**Variables** ^**a**^	**Total**		**HPV vaccine included in the National Immunization Program**		**HPV vaccine not included in the National Immunization Program**	
	***N* = 153 (100.0)**	**Range**	***n* = 97 (63.4)**	**Range**	***n* = 56 (36.6)**	**Range**
**Age (mean, SD, years), *n* = 139**	38.5 (10.1)	20–69	36.3 (9.2)	20–59	42.9 (10.4)	25–69
	*n* (%)	95% CI	*n* (%)	95% CI	n (%)	95% CI
**Gender**	**n = 139**					
Female	75 (54.0)	45.6–62.3	56 (61.5)	51.4–71.7	19 (39.6)	25.6–53.6
Male	64 (46.0)	37.7–54.4	35 (38.5)	28.3–48.6	29 (60.4)	46.4–74.4
**Country by region** ^**b**^	**n = 153**					
Eastern Africa	16 (10.5)	5.6–15.4	7 (7.2)	2.0–12.4	9 (16.1)	6.3–25.8
Middle Africa	64 (41.8)	33.9–49.7	52 (53.6)	43.6–63.6	12 (21.4)	10.6–32.3
Northern Africa	10 (6.5)	2.6–10.5	9 (9.3)	3.4–15.1	1 (1.8)	0.0–5.3
Southern Africa	6 (3.9)	0.8–7.0	6 (6.2)	1.3–11.0	0	-
Western Africa	57 (37.3)	29.5–45.0	23 (23.7)	15.2–32.3	34 (60.7)	47.8–73.7
**Provider**	**n = 134**					
Doctors/Residents	87 (64.9)	56.7–73.1	54 (60)	49.7–70.3	33 (75)	62.0–88.0
Nurses/Midwives	25 (18.7)	12–25.3	18 (20)	11.6–28.4	7 (15.9)	5.0–26.9
Other^c^	22 (16.4)	10.1–22.8	18 (20)	11.6–28.4	4 (9.1)	0.5–17.7
**Setting**	**n = 126**					
Rural	13 (10.3)	4.9–15.7	10 (11.4)	4.6–18.1	3 (7.9)	0.0–16.6
Urban	113 (89.7)	84.3–95.1	78 (88.6)	81.9–95.4	35 (92.1)	83.4–100.0

^a^For some variables, the total number of observations does not add up to 153 because of missing data. ^b^The UN Statistics Division has subdivided the African continent into five regions: Northern Africa (Algeria, Egypt, Libya, Morocco, Sudan, Tunisia, Western Sahara), Central or Middle Africa (Angola, Cameroon, Central African Republic, Chad, Equatorial Guinea, Congo, Democratic Republic of Congo, Gabon, Sao Tome and Principe), Southern Africa (Eswatini, Lesotho, Namibia, Botswana, South Africa), Eastern Africa (Eritrea, Djibouti, Somalia, Burundi, Rwanda, Uganda, South Sudan, Kenya, Tanzania, Seychelles, Comoros, Malawi, Madagascar, Mozambique, Zambia, Mauritius, Zimbabwe), and Western Africa (Burkina Faso, Benin, Togo, Ghana, Cote d’Ivoire, Liberia, Sierra Leone, Guinea, Guinea-Bissau, Senegal, Gambia, Cape Verde, Mauritania, Mali, Niger, Nigeria). ^c^The “other” category included health administrators, community health workers, and program managers.

### HPV vaccine availability and training of providers to perform HPV vaccine education

We assessed providers’ prior training on HPV vaccine education and the availability of the HPV vaccine in their practice according to the HPV vaccine national introduction status of the country where they work (Table 2). Up to 73 respondents (56.2%) indicated having received no prior training on HPV vaccine administration and related counseling; this proportion did not differ between countries with HPV NIPs and countries without HPV NIPs (54.1% versus 60.0%, *p* = 0.58) or between female providers compared to male providers (60.0% versus 51.7%, *p* = 0.38) (Supplementary Table S2). Only about one-third (40, 37.4%) of providers reported that the HPV vaccine was available at the facility where they work; this proportion was twice as high among participants in countries with HPV NIPs compared to those in countries without HPV NIPs (43.8% versus 23.5%, *p* = 0.05) (Table 2).

**Table 2:** Prior training and availability of HPV vaccine from a providers’ perspective in Africa according to the introduction status of HPV vaccine into the national immunization program.

**Variables** ^**a**^	**Total**		**Are you working in a country that has introduced HPV vaccine in the National Immunization Program?**						
			**Yes**			**No**			***p*-value** ^**b**^
	*N* (%)	**95% CI**	*N* (%)		**95% CI**	*N* (%)		**95% CI**	
**Have you previously had training in how to educate your patients, their family members, and the population about HPV vaccination?**									0.58
No	73 (56.2)	47.5–64.8	46 (54.1)		43.4–64.9	27 (60.0)		45.5–74.5	
Yes	57 (43.8)	35.2–52.5	39 (45.9)		35.1–56.6	18 (40.0)		25.5–54.5	
**My knowledge about HPV vaccination is adequate for my current practice**									0.06
Agree	63 (52.9)	43.8–62.0	43 (55.8)		44.6–67.1	20 (47.6)		32.3–62.9	
Disagree	36 (30.3)	21.9–38.6	18 (23.4)		13.8–33.0	18 (42.9)		27.7–58.0	
Neutral	20 (16.8)	10.0–23.6	16 (20.8)		11.6–30.0	4 (9.5)		0.5–18.5	
**Is HPV vaccine available at the facility where you work?**									**0.05**
No	67 (62.6)	53.3–71.9	41 (56.2)		44.6–67.7	26 (76.5)		62.0–91.0	
Yes	40 (37.4)	28.1–46.7	32 (43.8)		32.3–55.4	8 (23.5)		9.0–38.0	
**Do you currently recommend HPV vaccination in your practice?**									**0.05**
No	22 (16.8)	10.3–23.3	10 (11.8)		4.8–18.7	12 (26.1)		13.2–38.9	
Yes	109 (83.2)	76.7–89.7	75 (88.3)		81.3–95.2	34 (73.9)		61.1–86.8	
**Who do you recommend the HPV vaccine for?**									0.49
Girls only	87 (82.1)	74.7–89.5	62 (83.8)		75.2–92.3	25 (78.1)		63.6–92.7	
Girls and boys	19 (17.9)	10.5–25.3	12 (16.2)		7.7–24.8	7 (21.9)		7.3–36.4	

^a^For some variables, the total number of observations does not add up to 153 because of missing data. ^b^The *p*-value was calculated using the likelihood ratio chi-square test for continuous variables and the Fisher’s exact test for categorical variables.Bold values are highlight statistically significant *p*-values.

### HPV vaccine recommendation practices

A majority of providers (109, 83.2%) reported that they were recommending the HPV vaccine in their practice; this proportion was significantly higher among providers based in countries with HPV NIPs compared to those in countries without HPV NIPs (88.3% versus 73.9%, *p* = 0.05) (Table 2). The proportion of providers who reported recommending the HPV vaccine was higher among female providers compared to male providers (87.3% versus 78.3%), although the difference was not statistically significant (*p* = 0.17) (Supplementary Table S2).

Among providers who reported recommending the HPV vaccine in their practice, 87 (82.1%) recommended it for girls only (per the WHO guidelines), and 19 (17.9%) recommended it for both girls and boys (gender-neutral vaccination, per the US CDC guidelines). There was no significant difference in the proportion of providers who reported girl-only recommendation according to the HPV vaccine national introduction status of the country where they work (83.8% versus 78.1%, *p* = 0.49), and according to gender (78.7% versus 86.7%, *p* = 0.29).

**Figure 2:**
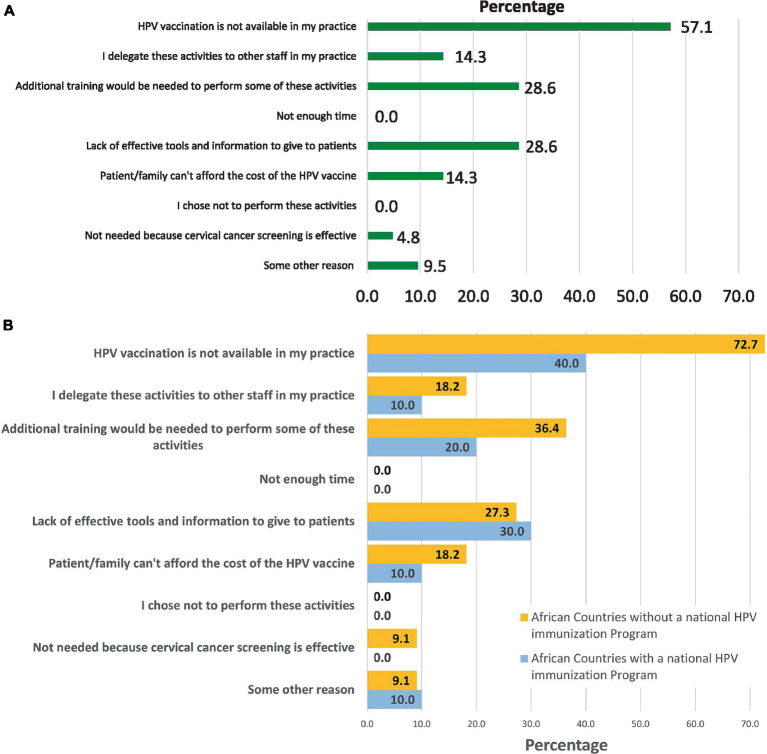
Reasons for not recommending HPV vaccination among providers involved in cervical cancer prevention activities in Africa. **(A)** Overall. The percentages add up to more than 100% because some respondents selected more than one reason for not recommending HPV vaccine in their practice. **(B)** According to the introduction status of HPV vaccine into the national immunization program. The percentages add up to more than 100% because some respondents selected more than one reason for not recommending HPV vaccine in their practice.

Among providers who did not recommend the HPV vaccine (16.8%), the most frequent reasons for not recommending it included: vaccine unavailability (57.1%), lack of effective tools and information to give to patients (28.6%), and need for additional training (28.6%) (Figure 2A). The proportion of providers who reported either of these reasons was higher among respondents based in countries without HPV NIPs compared to those based in countries with HPV NIPs (Figure 2B), and among male providers compared to female providers (Supplementary Figure S2).

### HPV vaccine knowledge among African healthcare providers

#### Perceived knowledge

Providers were also asked if their knowledge about HPV vaccination was adequate for their current practice. Of the 119 providers who responded to this question, 63 (52.9%) agreed with this statement; there was no difference between providers working in countries with HPV NIPs and those working in countries without HPV NIPs (55.8% versus 47.6%, *p* = 0.06) (Table 2), and between female providers and male providers (53.1% versus 52.7%, *p* = 0.45) (Supplementary Table S2).

#### Measured knowledge

Providers’ knowledge about the HPV vaccine was measured with three knowledge questions. Of the 121 providers who responded to the knowledge questions, 17 (14.0%) respondents provided the correct answer to the question about vaccine efficacy (question i); 32 (26.4%) provided the correct answer to the question about vaccine administration schedule (question ii); and 111 (91.7%) provided the correct answer to the question about vaccine safety (question iii). Overall, 9.9% of providers answered correctly to all three knowledge questions; this proportion was twice as high among providers based in countries with HPV NIPs compared to providers in countries without HPV NIPs (12.3% versus 5.0%, *p* = 0.34), and among female providers compared to male providers (13.4% versus 5.6%, *p* = 0.26), although the difference was not statistically significant.

## Discussion

In this study, we examined the training and knowledge of African healthcare providers involved in cervical cancer prevention activities, as well as their recommendation practices for the HPV vaccine. While a majority of providers reported recommending the HPV vaccine, their level of knowledge about this vaccine was relatively low. Further, most providers have not been trained in performing HPV vaccine education/counseling and reported that the HPV vaccine remains unavailable in their practice.

Provider’s recommendation of HPV vaccine has been examined in different regions of the world. In a survey conducted in China before the COVID-19 pandemic, 66.4% of HCPs reported recommending (either sometimes, usually, or always) HPV vaccination in their practice (18). In two recent surveys conducted during the COVID-19 pandemic, the proportion of HCPs who recommended HPV vaccination was 57% in Japan (19), and 59% in the State of New York in the US (20). The higher prevalence of HPV vaccine recommendation observed in our study sample is likely explained by our target population which consisted of HCPs involved in cervical cancer prevention activities, and thus, who were more actively engaged in addressing cervical cancer and HPV-related diseases in their practice.

Our findings suggest a relatively low level of training and knowledge about the HPV vaccine among healthcare providers in Africa, including in countries that have introduced HPV immunization in their NIPs. The poor compliance with HPV vaccination in many of these countries may be explained by a limited experience in implementing routine immunization programs for adolescents, as vaccines administered through NIPs in most African settings were primarily targeting newborns and/or infants (21). Corroborating previous reports (22), our findings suggest that adequate training to increase healthcare providers’ knowledge about the HPV vaccine will contribute to the success of HPV NIPs in Africa. Since adolescent immunization is not routinely practiced in many African countries, the introduction of HPV vaccines has created a unique opportunity to build platforms for adolescent immunization and related sexual and reproductive health services.

Vaccination is considered to be the most effective intervention for controlling infectious diseases and other vaccine-preventable diseases like cancers (23). However, the emergence of vaccine hesitancy, fueled by the COVID-19 pandemic, and its penetration into mainstream media may adversely affect the impact of immunization programs, especially in Africa, where access to the internet and social media networks are rapidly growing. Our study was conducted in the midst of the COVID-19 pandemic and provides insights about African providers’ HPV vaccination knowledge and recommendation against a backdrop of increased distrust toward the healthcare system and increased vaccine hesitancy (24). In many parts of Africa, inaccurate messages about HPV vaccine safety were often disseminated through social media (25). In a recent systematic review assessing barriers and facilitators of HPV vaccination in sub-Saharan Africa, limited health system capacities, socio-economic status, stigma, fear and costs of vaccines, negative experience with vaccinations, COVID-19 pandemic, lack of adequate information were identified as the main barriers (26). Further, the inadequate training of the workforce in Africa that was observed in this study, has emerged as the major constraint to the implementation of HPV vaccination programs in the region (27), and highlights the need for targeted interventions to improve providers’ education on this highly effective vaccine. There is strong evidence that training providers to improve their recommendation practices can be accomplished with evidence-based communication interventions (28). In our study, only one-fourth of providers responded correctly to the question about the vaccine administration schedule, suggesting that vaccine stock could be better utilized with prior appropriate training of providers and other stakeholders, especially in the context of a global shortage of HPV vaccines. The recent recommendation by the WHO of a single-dose regimen to achieve adequate protection against HPV-related diseases may further simplify the vaccine administration schedule, thereby facilitating the communication about the vaccine, and reducing the logistics for deploying and administering the vaccine, particularly to the population in remote areas. This may contribute to reducing disparities in access to the HPV vaccine in Africa.

A majority of African providers in this study reported recommending HPV vaccination only for girls per the WHO guidelines, as opposed to the gender-neutral strategy which has been adopted in most high-income countries. While the recently updated WHO recommendations for HPV vaccination remained focused on females, the single-dose strategy may provide an opportunity to introduce gender-neutral vaccination in high-burden settings like Africa, which may further accelerate herd immunity and the subsequent elimination of cervical cancer while reducing the growing burden of other HPV related diseases in the region (29). By removing the stigma associated with a female-only health intervention, the gender-neutral vaccination approach may improve population engagement and political involvement in a setting where community leaders and decision-makers are predominantly male. In our study, a gender-difference (although not statistically significant) was observed in the knowledge and recommendation practices for the HPV vaccine, with the proportion of providers who responded correctly to the knowledge questions and that of providers who reported recommending the HPV vaccine being higher among women compared to men.

Our study had some limitations. First, most respondents to the survey were clinicians working in urban settings, suggesting that our findings may not representatively reflect the status of HPV vaccine knowledge and recommendation practices among providers living in rural areas. Second, respondents were selected among participants in a continuing education e-learning program aimed at building the capacity of African providers in the field of cervical cancer prevention and management, and the survey was administered online. As a result, providers in areas with limited internet penetration were underrepresented in our study sample.

Our study reports pertinent data on provider HPV vaccine knowledge and recommendation against a backdrop of increasing vaccine hesitancy in the African population (24). Because provider recommendation is one of the major facilitators of vaccination uptake, culturally relevant programs should focus on reducing barriers and enhancing factors that promote providers’ recommendation of HPV vaccination. In developed country settings, relevant organizations have worked to increase the frequency and quality of HPV vaccination recommendations. The American Cancer Society has developed a guidebook for clinicians that uses evidence-based strategies to increase HPV vaccination (30). In the same way, African-based organizations, and immunization advisory bodies of African Ministries of Health should develop context-specific and evidence-based recommendations for increasing HPV vaccine recommendations by African providers. About a decade ago, a sub-Saharan African Cervical Cancer Working Group expert panel developed consensus recommendations for the prevention of cervical cancer in the region (including the importance of the HPV vaccine based on the latest clinical data) (31), such recommendations should be updated in the light of scientific advances and emerging needs of the African population.

## Conclusion

Despite a high rate of HPV vaccine recommendation among providers in Africa, provider training and knowledge about HPV vaccination remain suboptimal, and vaccine availability remains limited. To increase HPV vaccine uptake in a context of increased vaccine hesitancy in the region, further training is needed to improve providers’ knowledge and engagement. In addition, efforts to develop tailored educational material should be reinforced to facilitate culturally sensitive communication of providers with their patients and the community.

The recent recommendation by the WHO of a single-dose HPV vaccination regimen to prevent HPV-related diseases provides a unique opportunity for African countries to increase HPV vaccination coverage in an economically and logistically viable way. To that end, further training to improve healthcare providers’ knowledge about the HPV vaccine is needed in the region to enhance patient-provider communication, promote mass sensitization, and counter misinformation about the HPV vaccine. Context-specific and culturally sensitive educational tools and informational material to boost HPV vaccination in Africa should be developed.

## Data availability statement

The original contributions presented in the study are included in the article/Supplementary material, further inquiries can be directed to the corresponding author.

## Ethics statement

The studies involving humans were approved by the study protocol was approved by the University of Texas MD Anderson Cancer Center’s IRB. The studies were conducted in accordance with the local legislation and institutional requirements. The participants provided their written informed consent to participate in this study.

## Author contributions

JF: Conceptualization, Data curation, Formal analysis, Methodology, Project administration, Writing – original draft. ID: Conceptualization, Project administration, Writing – review & editing. SK: Writing – review & editing. RY: Data curation, Formal analysis, Methodology, Writing – review & editing. FG: Project administration, Writing – review & editing. LC: Writing – review & editing. GM: Writing – review & editing. NM: Project administration, Writing – review & editing. MPa: Data curation, Methodology, Supervision, Writing – review & editing. FS: Writing – review & editing. JK: Conceptualization, Methodology, Supervision, Writing – review & editing. BT: Project administration, Writing – review & editing. HE: Supervision, Writing – review & editing. MD: Supervision, Writing – review & editing. P-MT: Supervision, Writing – review & editing. MID: Supervision, Writing – review & editing. FL: Supervision, Writing – review & editing. IA: Conceptualization, Supervision, Writing – review & editing. MPl: Conceptualization, Supervision, Writing – review & editing. PB: Conceptualization, Supervision, Writing – review & editing. J-MD: Conceptualization, Supervision, Writing – original draft. SS: Conceptualization, Data curation, Formal analysis, Funding acquisition, Methodology, Project administration, Supervision, Writing – original draft.
