# Supplementary material for: HPV vaccination in Africa in the COVID-19 era: a cross-sectional survey of healthcare providers’ knowledge, training, and recommendation practices
Source: Front Public Health. 2024 Jan 17;12:1343064. doi: 10.3389/fpubh.2024.1343064 (PMC10829043; doi:10.3389/fpubh.2024.1343064)
Supplement: Supplementary file 1 [file Data_Sheet_1.docx]

**Supplemental Material**

**Supplemental Table 1: STROBE STATEMENT Checklist for Cross-Sectional Studies**

|  | Item No | Recommendation |  |
| --- | --- | --- | --- |
| **Title and abstract** | 1 | (*a*) Indicate the study’s design with a commonly used term in the title or the abstract |  |
|  |  | (*b*) Provide in the abstract an informative and balanced summary of what was done and what was found |  |
| Introduction | | |  |
| Background/rationale | 2 | Explain the scientific background and rationale for the investigation being reported |  |
| Objectives | 3 | State specific objectives, including any prespecified hypotheses |  |
| Methods | | |  |
| Study design | 4 | Present key elements of study design early in the paper |  |
| Setting | 5 | Describe the setting, locations, and relevant dates, including periods of recruitment, exposure, follow-up, and data collection |  |
| Participants | 6 | (*a*) Give the eligibility criteria, and the sources and methods of selection of participants |  |
| Variables | 7 | Clearly define all outcomes, exposures, predictors, potential confounders, and effect modifiers. Give diagnostic criteria, if applicable |  |
| Data sources/ measurement | 8* | For each variable of interest, give sources of data and details of methods of assessment (measurement). Describe comparability of assessment methods if there is more than one group |  |
| Bias | 9 | Describe any efforts to address potential sources of bias |  |
| Study size | 10 | Explain how the study size was arrived at |  |
| Quantitative variables | 11 | Explain how quantitative variables were handled in the analyses. If applicable, describe which groupings were chosen and why |  |
| Statistical methods | 12 | (*a*) Describe all statistical methods, including those used to control for confounding |  |
|  |  | (*b*) Describe any methods used to examine subgroups and interactions |  |
|  |  | (*c*) Explain how missing data were addressed |  |
|  |  | (*d*) If applicable, describe analytical methods taking account of sampling strategy |  |
|  |  | (*e*) Describe any sensitivity analyses |  |
| Results | | |  |
| Participants | 13* | (a) Report numbers of individuals at each stage of study—eg numbers potentially eligible, examined for eligibility, confirmed eligible, included in the study, completing follow-up, and analysed | |
|  |  | (b) Give reasons for non-participation at each stage | |
|  |  | (c) Consider use of a flow diagram | |
| Descriptive data | 14* | (a) Give characteristics of study participants (eg demographic, clinical, social) and information on exposures and potential confounders | |
|  |  | (b) Indicate number of participants with missing data for each variable of interest | |
| Outcome data | 15* | Report numbers of outcome events or summary measures | |
| Main results | 16 | (*a*) Give unadjusted estimates and, if applicable, confounder-adjusted estimates and their precision (eg, 95% confidence interval). Make clear which confounders were adjusted for and why they were included | |
|  |  | (*b*) Report category boundaries when continuous variables were categorized | |
|  |  | (*c*) If relevant, consider translating estimates of relative risk into absolute risk for a meaningful time period |  |
| Other analyses | 17 | Report other analyses done—eg analyses of subgroups and interactions, and sensitivity analyses |  |
| Discussion | | |  |
| Key results | 18 | Summarize key results with reference to study objectives |  |
| Limitations | 19 | Discuss limitations of the study, taking into account sources of potential bias or imprecision. Discuss both direction and magnitude of any potential bias |  |
| Interpretation | 20 | Give a cautious overall interpretation of results considering objectives, limitations, multiplicity of analyses, results from similar studies, and other relevant evidence |  |
| Generalizability | 21 | Discuss the generalizability (external validity) of the study results |  |
| Other information | | |  |
| Funding | 22 | Give the source of funding and the role of the funders for the present study and, if applicable, for the original study on which the present article is based |  |

**Supplemental Table 2**: **Prior training and availability of HPV vaccine from a providers’ perspective in Africa** **according to gender**

| **Variable** | **Total** | | **Gender** | | | | | |  |
| --- | --- | --- | --- | --- | --- | --- | --- | --- | --- |
|  |  |  | **Female** | | | **Male** | | | **p-value** |
|  | N (%) | **95% CI** | N (%) | | **95% CI** | N (%) | | **95% CI** |  |
| **Have you previously had training in how to educate your patients, their family members, and the population about HPV vaccination?** | | |  |  |  |  |  |  | 0.38 |
| No | 73 (56.2) | 47.5 – 64.8 | 42 (60.0) | | 48.4 – 71.6 | 31 (51.7) | | 38.9 – 64.5 |  |
| Yes | 57 (43.8) | 35.2 – 52.5 | 28 (40.0) | | 28.4 – 51.6 | 29 (48.3) | | 35.5 – 61.1 |  |
| **My knowledge about HPV vaccination is adequate for my current practice** | | |  | |  |  | |  | 0.45 |
| Agree | 63 (52.9) | 43.8 – 62.0 | 34 (53.1) | | 40.7 – 65.5 | 29 (52.7) | | 39.3 – 66.1 |  |
| Disagree | 36 (30.3) | 21.9 – 38.6 | 17 (26.6) | | 15.6 – 37.5 | 19 (34.5) | | 21.8 – 47.3 |  |
| Neutral | 20 (16.8) | 10.0 – 23.6 | 13 (20.3) | | 10.3 – 30.3 | 7 (12.7) | | 3.8 – 21.7 |  |
| **Is HPV vaccine available at the facility where you work?** | | |  |  |  |  |  |  | 0.30 |
| No | 67 (62.6) | 53.3 – 71.9 | 35 (58.3) | | 45.7 – 71.0 | 32 (68.1) | | 54.5 – 81.6 |  |
| Yes | 40 (37.4) | 28.1 – 46.7 | 25 (41.7) | | 29.0 – 54.3 | 15 (31.9) | | 18.4 – 45.5 |  |
| **Do you currently recommend HPV vaccination in your practice?** | | |  |  |  |  |  |  | 0.17 |
| No | 22 (16.8) | 10.3 – 23.3 | 9 (12.7) | | 4.8 – 20.5 | 13 (21.7) | | 11.1 – 32.2 |  |
| Yes | 109 (83.2) | 76.7 – 89.7 | 62 (87.3) | | 79.5 – 95.2 | 47 (78.3) | | 67.8 – 88.9 |  |
| **Who do you recommend the HPV vaccine for?** | | |  |  |  |  |  |  | 0.29 |
| Girls only | 87 (82.1) | 74.7 – 89.5 | 48 (78.7) | | 68.2 – 89.1 | 39 (86.7) | | 76.6 – 96.8 |  |
| Girls and boys | 19 (17.9) | 10.5 - 25.3 | 13 (21.3) | | 10.9 – 31.8 | 6 (13.3) | | 3.2 – 23.4 |  |

^a^ For some variables, the total number of observations does not add up to 153 because of missing data.

^b^The p-value was calculated using the likelihood ratio chi-square test for continuous variables and the Fisher’s exact test for categorical variables

**Supplemental Figure 1: Study Questionnaire**

**Provider’s training and recommendation of HPV vaccine**

**Supplemental Figure 2**: **Reasons for not recommending HPV vaccination among providers involved in cervical cancer prevention activities in Africa according to gender***


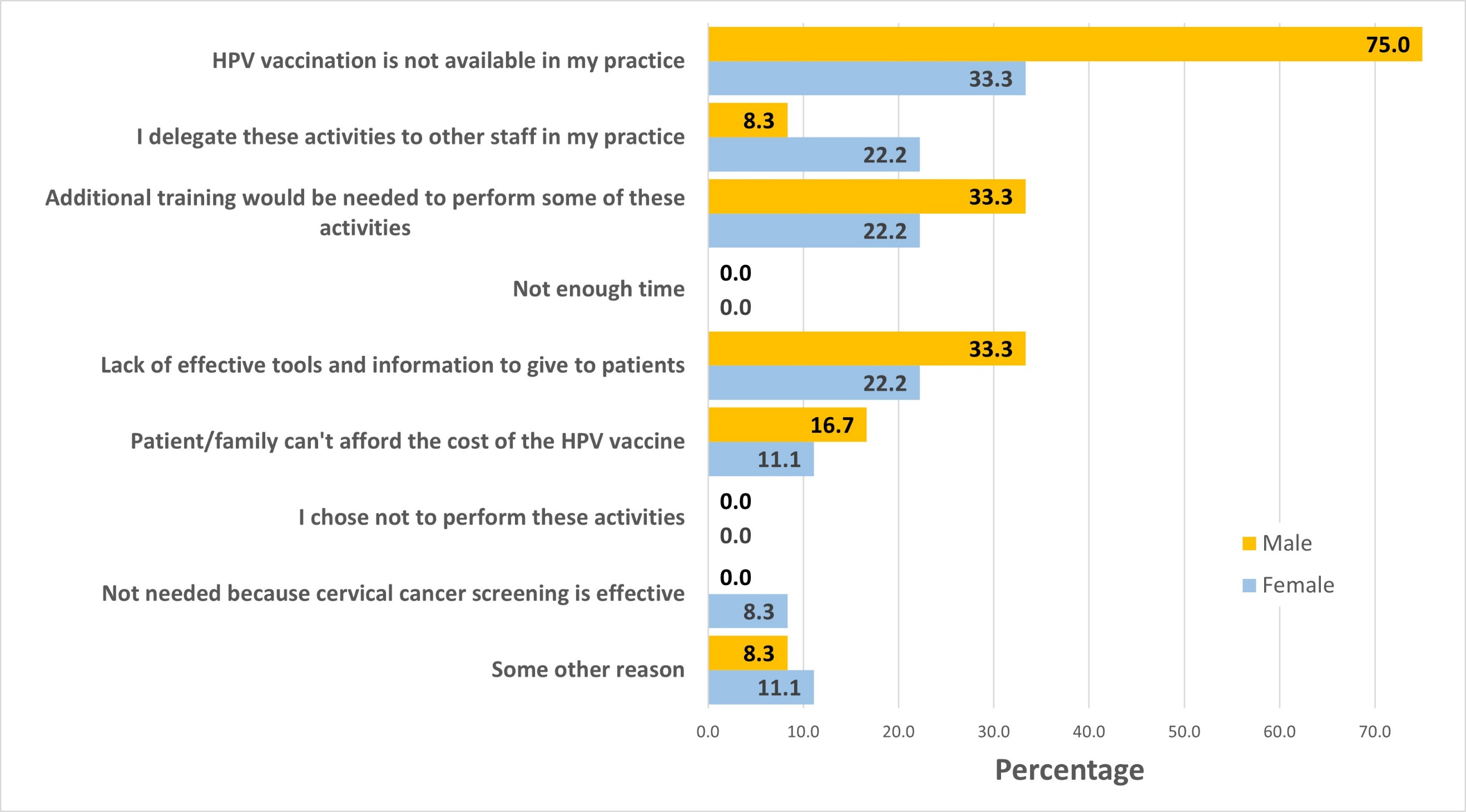


***The percentages add up to more than 100% because some respondents selected more than one reason for not recommending HPV vaccine in their practice**
